# Special Issue: A, B and Z: The Structure, Function and Genetics of Z-DNA and Z-RNA

**DOI:** 10.3390/ijms22147686

**Published:** 2021-07-19

**Authors:** Alan Herbert, Sergey Karapetyan, Maria Poptsova, Karen M. Vasquez, Quentin Vicens, Beat Vögeli

**Affiliations:** 1 InsideOutBio, Discovery, 42 2 8th Street, Unit 3412, Charlestown, MA 02129, USA; 2 External Communications Unit, Faculty of Computer Science, National Research University Higher School of Economics, 11 Pokrovsky Boulvar, 101000 Moscow, Russia; skarapetyan@hse.ru; 3 Laboratory of Bioinformatics, Faculty of Computer Science, National Research University Higher School of Economics, 11 Pokrovsky Boulvar, 101000 Moscow, Russia; mPoptsova@hse.ru; 4 Division of Pharmacology and Toxicology, Dell Pediatric Research Institute, College of Pharmacy, The University of Texas at Austin, 1400 Barbara Jordan Blvd, Austin, TX 78723, USA; karen.vasquez@austin.utexas.edu; 5 Department of Biochemistry and Molecular Genetics, University of Colorado Anschutz Medical Campus, Aurora, CO 80045, USA; quentin.vicens@cuanschutz.edu (Q.V.); beat.VOGELI@cuanschutz.edu (B.V.); 6 RNA BioScience Initiative, University of Colorado Anschutz Medical Campus, Aurora, CO 80045, USA

## Abstract

It is now difficult to believe that a biological function for the left-handed Z-DNA and Z-RNA conformations was once controversial. The papers in this Special Issue, “Z-DNA and Z-RNA: from Physical Structure to Biological Function”, are based on presentations at the ABZ2021 meeting that was held virtually on 19 May 2021 and provide evidence for several biological functions of these structures. The first of its kind, this international conference gathered over 200 scientists from many disciplines to specifically address progress in research involving Z-DNA and Z-RNA. These high-energy left-handed conformers of B-DNA and A-RNA are associated with biological functions and disease outcomes, as evidenced from both mouse and human genetic studies. These alternative structures, referred to as “flipons”, form under physiological conditions, regulate type I interferon responses and induce necroptosis during viral infection. They can also stimulate genetic instability, resulting in adaptive evolution and diseases such as cancer. The meeting featured cutting-edge science that was, for the most part, unpublished. We plan for the ABZ meeting to reconvene in 2022.

## 1. Introduction

Historically, the biological role of alternative nucleic acid structures has been controversial, especially for Z-DNA. The Z-DNA helix, with the phosphate backbone zig-zagging left rather than to the right in B-DNA and with its bases flipped over as well, was a surprise finding when the first synthetic DNA was crystalized in 1979 [1]. The structure solved was an answer to a question no one was asking. While the existence of left-handed DNA had been proposed in 1972 [2], biologists were stunned by the discovery. After the initial excitement, with abundant speculation as to its biological role being proposed, nothing immediately was found that was reproducible and the field was perceived by many as a dead-end [3]. The physical chemists, however, remained intrigued by the dynamic nature of this structure. Cell biologists focused on Z-DNA as a cause of genomic instability leading to cancer [4]. The larger community simply forgot about Z-DNA to the point that even this year it was necessary to remind the journal *Science* that Z-DNA is not a right-handed helix that incorporates the diaminopurine “z” base [5].

The unveiling of a biological role for the Z-conformation required new approaches to identify structure-specific proteins that recognized the left-handed structure and genetic studies to characterize the effects of variants on phenotypes. The first protein identified that was specific for Z-DNA was the double-stranded RNA editing enzyme ADAR1, which recognized Z-DNA and Z-RNA through its Zα domain. The interaction is structure-specific with no base-specific contacts and is of nanomolar affinity. The Zα domain was also found in another protein called Z-DNA binding protein 1 (ZBP1) and also in vaccinia viral proteins like E3L [6]. Human Mendelian genetic studies of Zα variants proved unambiguously that the Z-conformation was essential to the negative regulation of type I interferon responses [7], while the Zα sensed Z-RNA to induce the programmed cell death pathway called necroptosis during influenza infection [8]. These advances were informed by biochemical, biophysical and structural approaches that established the conditions and base modifications necessary to flip from the right- to left-handed conformation under physiological conditions [9]. Computational analyses established that the localization of the repeats that form Z-DNA is not random [10], consistent with its selection for or against particular outcomes.

This inaugural ABZ meeting (https://abz2021.bio, accessed 16 July 2021) was convened to bring together the leading researchers within the different disciplines that study the Z-conformation, with an intent to celebrate and collaborate. This field, now 50 years old [1,2], has had many recent successes that were presented at the conference. Some of these will feature in this Special Issue.

## 2. Meeting Summary

Over 200 participants from 24 different countries spread across 5 continents participated. They were drawn from many diverse fields, including chemistry, biophysics, biochemistry, structural biology, genetics, immunology, cell biology and virology. A total of eighteen speakers presented in three main sessions, with a deliberate focus on presenting unpublished data. For that reason, formal abstracts were not requested. There were no poster sessions given the one-day format and the many time zones.

A number of themes emerged from the presentations and follow-up discussions. Based on presentations by Yukio Kawahara [11], Alan Herbert [12], Tony Sun [13] and Qingde Wang [14], the key role of ADAR1 p150, which contains the Z-DNA and Z-RNA binding Zα domain in regulating type I interferon responses in both human and mice, is now beyond doubt. The Kawahara lab has constructed a mouse model that expresses the ADAR p150 isoform that contains the Zα domain, but not the shorter p110 isoform. The p150 mice were normal without any dysregulation of interferon responses but required an editing null p110 allele to thrive in the period following weaning. Tony Sun presented constructs that expressed p150 but not p110 and demonstrated p150-specific editing. Qingde Wang presented a mouse model that had a severe neurological phenotype associated with a p150 loss of function Zα variant paired with a Zα null allele, resembling that seen in Aicardi-Goutières disease in humans. This model has promise for understanding the critical role played by ADAR1 p150 in preventing neuronal cell death and may provide insight on cell death pathways in other neurological diseases.

The key role of the related ZBP1 in the inflammatory cell death called “necroptosis” is also clearly established for viral infections due to vaccinia (Bert Jacobs and Ed Mocarski [15]) and influenza (Sid Balachandran [8] and Thirumala-Devi Kanneganti [16]). Studies showed a key role for both the Zα and receptor-interacting serine/threonine kinase homotypic interaction motif (RHIM) domains in the induction of necroptosis during influenza infections and that the vaccinia E3L protein inhibits this action of ZBP1. The Zα RHIM have been previously shown to activate other inflammatory pathways that do not involve cell death [17,18]. How these findings apply to chronic diseases in the clinic was addressed by David Pisetsky [19]. He presented data showing that many of the DNA binding antibodies, and specifically those for Z-DNA, found in patients with systemic lupus erythematosus, bind to the DNAs of many common pathogens, especially those like *M. tuberculosis* that have a high guanine/cytosine content in their genome.

Manolis Pasparakis presented genetic models identifying pathways in which endogenous ZBP1 dsRNA substrates are associated with disease outcomes in the gut and the skin [20]. In particular, when other cell death pathways are inactivated by mutation, necroptosis that is dependent on ZBP1 produces inflammation and disease due to necroptosis. Other labs (Jon Maelfait [21], Jan Rehwinkel [22] and Andrew Oberst (unpublished) presented studies from mouse models to examine the genetic interactions between ZBP1, MDA5 and ADAR1 and their outcomes, in particular on live births. Preliminary results were presented showing that in certain crosses, embryonic lethality was associated with high interferon responses in the mother but not dependent on the paternal genotype. The sequences targeted by ADAR1 and ZBP1 identified in mice that expressed ADAR1 p150 do not currently map to any one set of proteins or pathways.

Maria Poptsova [10] presented computational approaches based on the DeepZ machine learning algorithms to study factors that alter the B to Z flip energetics in vivo and to describe the pathways involved. Structural studies on Z-RNA formation by Quentin Vicens [23] and Beat Vögeli [9] outlined some of the different requirements for Z-RNA formation compared to Z-DNA formation, pointing at a widespread distribution of Z-RNA across transcriptomes. Their work shows that the rules for Z-RNA formation differ from those for Z-DNA formation. In particular, bubbles and bulges in double-stranded RNA decrease the energetic cost of forming A-RNA/Z-RNA junctions, while non-canonical base pairs like those formed by guanine paired to uracil also enable the flip. Elegant single-molecule studies were presented by Seok-Cheol Hong [24] and Zev Bryant [25], providing precise details for the flip from right- to left-handed structures and how these approaches enable future studies of the molecular machines involved in these processes.

Expanding on these themes, the evolutionary roles of simple repeat sequences that adopt different conformations under physiological conditions, called “flipons”, was presented by Alan Herbert [26]. These sequences act as switches to change biological outcomes. He presented a model in which the helicase MDA5 acts on host transcripts to induce Z-RNA formation by Alu inverted repeat elements. The flip from A-RNA to Z-RNA then localizes ADAR p150 to these RNAs and initiates editing that prevents activation of interferon responses against self while allowing those against viral sequences that lack these elements to proceed. This model suggests that there may be active selection for Z-forming Alu elements in the human genome. Karen Vasquez [27] examined the mechanistic processing of mutagenic H-DNA and Z-DNA by DNA repair proteins that maintain genomic integrity. When repair fails, these rearrangements may lead to cancer or speed evolutionary adaptation, as evidenced by studies of an enhancer element in stickleback fish that determines whether pelvic hind fins form [28].

The breakout rooms proved a great success allowing many colleagues who have contributed to the field over the years to reconnect and exchange recollections with those new to this endeavor. Tom Jovin, Ron Hill, Shuguang Zhang and Horace Drew anchored the historical discussion, with Guliang Wang, Cat Musselman, and Heather Koehler moderating rooms focusing on the structure, biology and genetics of all things Z.

## 3. Conclusions

As co-organizers of this online event, we were thrilled by the many recently published and unpublished results that were presented, attesting to the vibrancy of the field. We were also pleased by the surprise expressed by many in the field about how much they were previously unaware of, even though many have been working on one aspect or another of Z-DNA and Z-RNA for many years. It is now clear how complex history can be, with many key discoveries occurring out of sequence with their biological significance not fully understood until years later. During a social hour with theme-designated breakout rooms, many new collaborations were discussed, further indicating the ABZ2021 meeting served its purpose to further stimulate the field. With many exciting new developments, the meeting participants enthusiastically endorsed an annual ABZ meeting. We hope to capture all this excitement in the papers within this Special Issue and pave the way for the many new discoveries to come.

## 4. Future Directions

As with any rapidly developing field, there are always more questions than answers. While studies of the Zα domain have enabled much of the impressive progress made so far in understanding the biological roles of Z-DNA and Z-RNA, the question is whether there are other Z-binding domain families that regulate other important biological outcomes such as chromatin structure, RNA splicing and RNA 3′ UTR. If so, are these domains exclusively Z-specific rather than also binding to other conformations. For instance, a protein could bind both B-DNA and Z-DNA under different conditions. This outcome would be analogous to that seen for the telomere binding Rap1 protein of yeast. Rap 1 binds both B-DNA and to a G4 quadruplex through different faces of the same helix [29]. From a computational genetics perspective, there are existing programs to predict Z-DNA formation by naked DNA. There is a need to develop algorithms for predicting Z-RNA forming sequences and also for identifying epigenetic factors influencing Z-formation in vivo. Further, we do not know whether the Z-forming substrates involved in ADAR1 and ZBP1 pathways are the same. If not, do they differ by repeat family or by the conditions under which these sequences are expressed? Is ZBP1 activation involved in neurological diseases like AGS due to ADAR loss of function variants? Does ZBP1 drive the necroptosis present in some forms of Alzheimer’s Disease [30]? Future studies will be helped by drugs that can alter z-flipon conformation. An important question is whether drugs that affect Z-formation have a therapeutic application, for example, in activating cancer cell death by ZBP1 or by inhibiting necroptosis in normal tissues? As the field grows, many others may find that they have been unwittingly working with alternative nucleic acid conformations like Z-DNA and Z-RNA all along. They will be like Molière’s Monsieur Jourdain, who found, much to his surprise, that he had “been speaking prose while knowing nothing of it”.
